# Anti-Inflammatory Effect of Curcumin on the Mouse Model of Myocardial Infarction through Regulating Macrophage Polarization

**DOI:** 10.1155/2021/9976912

**Published:** 2021-08-21

**Authors:** Shaoxi Yan, Mo Zhou, Xiaoyun Zheng, Yuanyuan Xing, Juan Dong, Mengwen Yan, Rui Li

**Affiliations:** ^1^Department of Health Care, China-Japan Friendship Hospital, Ministry of Health, China; ^2^Department of Cardiology, China-Japan Friendship Hospital, China

## Abstract

Inflammation causes tissue damage and promotes ventricular remodeling after myocardial infarction (MI), and the infiltration and polarization of macrophages play an important role in regulating inflammation post-MI. Here, we investigated the anti-inflammatory function of curcumin after MI and studied its relationship with macrophage polarization. In vivo, curcumin not only attenuated ventricular remodeling 3 months after MI but also suppressed inflammation during the first 7 days post-MI. Importantly, the results of qPCR and immunochemistry showed that curcumin decreased M1 (iNOS, CCL2, and CD86) but increased M2 macrophage (Arg1, CD163, and CD206) marker expression in the myocardium of MI mice during the first 7 days post-MI. And flow cytometry analysis indicated that curcumin suppressed M1 (CD45+Gr-1-CD11b+iNOS+ cells) but enhanced M2 macrophage (CD45+Gr-1-CD11b+Arg+ cells) expansion in the myocardium of MI mice during the first 7 days post-MI. In vitro, curcumin decreased LPS/IFN*γ*-elevated M1 macrophage marker (iNOS and CD86) expression and the proportion of M1 macrophages (iNOS+F4/80+ cells) but increased LPS/IFN*γ*-suppressed M2 macrophage marker (Arg1 and CD206) expression and the proportion of M2 macrophages (Arg1+F4/80+ cells). In addition, curcumin modulates M1/M2 macrophage polarization partly via AMPK. In conclusion, curcumin suppressed the MI-induced inflammation by modulating macrophage polarization partly via the AMPK pathway.

## 1. Introduction

Myocardial infarction (MI) is myocardial necrosis caused by acute and persistent ischemia and hypoxia of the coronary arteries, and about 1.5 million people in the United States suffer from MI each year, while China has 500,000 [1, 2]. In recent years, chest pain centers have been established in Chinese hospitals, which has enabled MI patients to receive timely treatment, which ultimately led to a significant reduction in the mortality of MI patients [2]. However, the prevalence of myocardial failure and arrhythmia caused by left ventricular remodeling after MI increases, which brings a huge socioeconomic burden [3]. Therefore, it is very meaningful to attenuate ventricular remodeling after myocardial infarction.

Myocardial tissue remodeling post-MI is a very complex physiological process, involving immunity, inflammation, extracellular matrix, immune cells, cardiomyocytes, and noncardiomyocytes (fibroblasts, smooth muscle cells, and endothelial cells). Myocardial inflammation caused by ischemia is the key to repair a damaged myocardium, but persistent inflammation is the main factor leading to myocardial remodeling and causing impaired cardiac function [4, 5]. Macrophages are recruited into the heart tissue after ischemia, M1 macrophages that exhibit proinflammatory effects dominate in the early stage of ischemia [6, 7], and then M2 macrophages that play a role in tissue repair function dominate during the subsequent period of inflammation remission [8, 9]. M1 macrophages have been found to be an important component of ventricular remodeling, leading to dilation and impaired function of the left ventricle through proinflammatory effects [10, 11]. Therefore, many scholars have emphasized that attenuating inflammation induced the ventricular remodeling by regulating the polarization of macrophages after myocardial infarction.

Curcumin, an active ingredient extracted from natural plants, has been proven to have many physiological effects, including hypolipidemic, antitumor, anti-inflammatory, and antioxidation, and was used as treatment for drug-resistant tuberculosis, etc. [12]. Importantly, previous studies have shown that curcumin promotes heart repair after myocardial infarction and improves cardiac dysfunction [13], as well as improving the therapeutic efficacy of heart failure after myocardial infarction [14], but the specific mechanism is imperfect. In addition, curcumin has been found to suppress inflammation by inhibiting macrophage infiltration [15], and it can also regulate macrophage polarization [16, 17]. In this study, we found that curcumin not only attenuated ventricular remodeling and inflammation after MI but also reduced M1 but increased M2 macrophage activation in the MI mouse model. In addition, in vitro studies also revealed that some curcumin regulates the molecular mechanism of macrophage polarization, which was regulated AMPK expression. All in all, our research enriches the mechanism of curcumin in the treatment of myocardial infarction.

## 2. Materials and Methods

### 2.1. Mouse and MI Model

A total of 105 C57BL/6 mice (9-12 weeks) were studied in the present study: 12 mice were used as wild-type (WT) or sham operation controls, 33 mice were used to build MI models without any treatment, 33 mice were used to build MI models treated with curcumin (78246, Merck, USA), and 27 mice were used to prepare bone marrow cells.

We established the MI mouse model as described below: mice were anesthetized with ketamine (50 mg/kg) and pentobarbital sodium (50 mg/kg). After its righting reflex had disappeared, we opened the thoracic cavity of the mouse and then quickly find the left coronary artery (LCA) under a microscope and then permanently ligated it with nylon suture at the site of the LCA (mice in the sham group were not ligated). The myocardial blanching was used as a reference to confirm the complete closure of blood vessels, and we excluded mice that failed to recover from anesthesia from the study. Mice in different groups are treated as follows: mice in the MI+Cur group received 100 mg/kg/day curcumin (78246, Merck, USA) for 6 weeks via intraperitoneal injection, and mice in the sham or MI group received equal volume of PBS via intraperitoneal injection. In addition, all animal experiments were approved by the Institutional Animal Experiment Committee of China-Japan Friendship Hospital and conformed to the NIH Guide for the Care and Use of Laboratory Animals.

### 2.2. Survival Analysis

A total of 30 MI mice were observed for 3 months of survival (15 mice in the MI group and 15 mice in the MI+Cur group), and it should be pointed out that we set a humane euthanasia endpoint for animal welfare, including a decrease in body temperature, reduced exercise, changes in breathing, and weight loss (>20%). When the above humane euthanasia endpoint appeared, we euthanized mice by intraperitoneal injection of 200 mg/kg pentobarbital sodium.

### 2.3. Histopathology and Immunohistochemistry

The mouse heart fixed with paraformaldehyde was made into a paraffin section. After dewaxing and hydration, paraffin sections are incubated with proteinase K to permeate cells. Next, we used a Masson three-color staining solution kit (G1340, Solarbio, China) as described in the product manufacturing instructions.

For the detection of protein expression using immunohistochemistry, a paraffin slice was taken and cut into 3.5 *μ*m slices by using a slicer, we put the slices in an autoclave, and they were heated to boiling and maintained for 3 minutes to repair the antigen. And then, slices were placed at 60°C for 2 h, deparaffinized, and hydrated with xylene and ethanol followed by PBS and double-distilled water to wash the retrieved nuclear antigen. Slices were first incubated with primary antibodies at 4°C overnight: anti-iNOS antibody (1 : 500, 13120, CST, USA) and anti-Arg1 antibody (1 : 500, 93668, CST, USA), and then incubated with HRP-goat anti-rabbit antibody (1 : 3000, ab205718, Abcam, UK). In addition, all slides (the nucleus) were counterstained with 5 *μ*g/mL DAPI for 5 minutes at room temperature.

### 2.4. Echocardiography

At 3 months post-MI, we used a Vevo 2100 instrument (Fujifilm, Japan) equipped with an MS-400 imaging transducer to detect echocardiography in mice as previously described [18].

### 2.5. Real-Time Quantitative PCR

Tissues were added to liquid nitrogen to break into small particles, and then, RNAiso Plus (9109, Takara, Japan) was used for cell lysis. And cells were harvested by centrifugation, and we used RNAiso Plus (9109, Takara, Japan) to lyse cells. At last, phenol chloroform/isopropanol was used to extract total RNA from cells. After preparing the cDNA using a PrimeScript RT reagent Kit with a gDNA eraser (RR047A, Takara, Japan), 20 *μ*L of the qPCR system was prepared and analyzed as described in the instructions of GoTaq qPCR Master Mix (A6001, Promega, USA). At last, we used the 2^−ΔΔCt^ method to analyze the relative gene expression, and *β*-actin was used as the loading control. The sequences of qPCR primers are shown in Table 1.

### 2.6. Cytokine Analysis

The tissue was homogenized using a MagNA Lyser tissue homogenizer (Roche, Switzerland) with cell culture medium and then centrifuged (1000 × *g*, 10 minutes, 4°C) to collect the supernatant. BMMs were treated with curcumin (10 *μ*mol/L) for 1 hour and then incubated with 100 mg/mL LPS+20 ng/mL IFN*γ* for 24 hours, and we directly collected cell culture medium to test cytokine. At last, we used the enzyme-linked immunosorbent assay kit to detect the cytokine concentration in the tissue homogenate and cell culture medium as described in the manufacturing instructions.

### 2.7. Heart Infiltrating Cell Isolation and Flow Cytometry Analysis

As previously described [19], we prepared cardiac single-cell suspensions. After removing the red blood cells and counting the cells, the single-cell suspensions were incubated for 30 min on ice with the following antibodies: anti-CD45 (Pacific Blue, 50-113-811, Invitrogen, USA), anti-CD11b (APC-Cy7, MABF512MI, MilliporeSigma, USA), and anti-Gr-1 (APC, RM3005, Invitrogen, USA). We performed intracellular staining after cells were fixed and permeabilized, i.e., incubation with following antibodies for 30 min on ice: anti-iNOS (PE, 14792, Cell Signaling Technology, USA) and anti-arginase 1 (FITC, 554001, BD, USA). Flow cytometry was used to detect the fluorescence, and data was analyzed by using FlowJo 7.6.2.

### 2.8. Preparation and Experiment of the Bone Marrow Macrophage

Mouse bone marrow macrophages (BMMs) were prepared and cultured according to previously described methods [20]. Briefly, isolate the femur and tibia of the mouse after anesthesia, cut off both ends of the bone, and rinse with cell culture, and then, centrifuge (500 × *g*, 10 minutes, 4°C) to collect bone marrow cells (BMs). Next, we used 10 ng/mL recombinant mouse macrophage colony-stimulating factor (cyt-439, AmyJet Scientific, China) to induce BMs to differentiate into macrophages (BMMs). In the present study, BMMs experienced stimulation with different stimuli: (1) BMMs were treated with curcumin (10 *μ*mol/L) for 1 hour and then incubated with 100 mg/mL LPS (L2630, Merck, USA)+20 ng/mL IFN*γ* (I4777, Merck, USA) for 24 hours, and (2) BMMs were treated with 10 *μ*mol/L Compound C (CC, HY-13418A, MCE, USA) for 2 hours and then incubated with curcumin (10 *μ*mol/L) for 1 hour, followed by 100 mg/mL LPS+20 ng/mL IFN*γ* challenge for 24 hours.

### 2.9. Immunoblotting

A total of 40 *μ*g tissue protein extract and cell protein extract was separated by 10% SDS-PAGE and then transferred to the PVDF membrane (IPVH00010, Millipore, USA). After blocking with 5% skimmed milk powder at room temperature for 2 hours, we incubated the PVDF membrane with primary antibodies at 4°C overnight: anti-iNOS antibody (1 : 1000, 13120, CST, USA), anti-CD86 antibody (1 : 1000, 91882, CST, USA), anti-Arg1 antibody (1 : 1500, 93668, CST, USA), anti-CD163 antibody (1 : 1000, ab182422, Abcam, UK), and *β*-actin (1 : 5000, ab8226, Abcam, UK). Next, the PVDF membranes were incubated with the HRP-goat anti-rabbit antibody (1 : 3000, ab205718, Abcam, UK) or HRP-goat anti-mouse antibody (1 : 3000, ab205719, Abcam, UK) at room temperature for 1 hour. At last, PVDF membranes were visualized with ECL solution (WBKLS0100, Beijing Xinjingke Biotechnologies Co., Ltd., China), followed by densitometry analysis using ImageJ 3.0 (IBM, USA), and *β*-actin was used as the loading control.

### 2.10. Statistical Analysis

GraphPad Prism 5 software was used for statistical analysis of data and drawing figures in the present study. Difference between two groups was compared by Student's *t*-test, and one-way ANOVA with the Tukey test as the post hoc test was used to compare the differences between multiple groups. The log-rank test was used to compare the survival of MI with or without curcumin treatment. *P* value less than 0.05 indicated significant difference.

## 3. Results

### 3.1. Curcumin Attenuates Ventricular Remodeling 3 Months after MI

**Figure 1:**
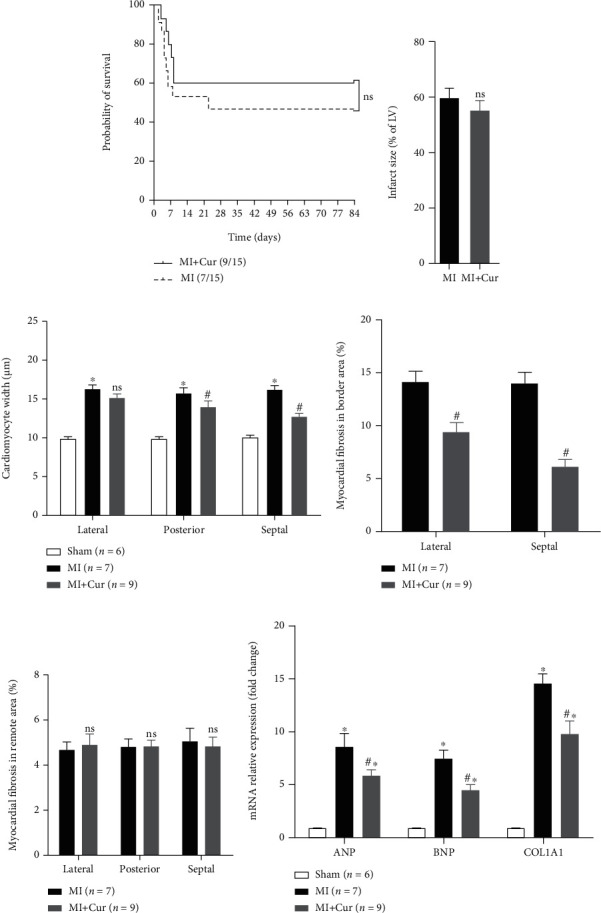
Curcumin attenuates adverse remodeling after MI: (a) 3-month overall survival analysis after MI; (b) quantitative analysis of infract size; (c) cardiomyocyte width; (d) myocardial fibrosis in border; (e) remote area; (f) qPCR analysis of ANP, BNP, and COL1A1 mRNA expression in the myocardium at 3 months post-MI. Data were shown as the mean ± SEM. ∗ was *P* < 0.05 vs. sham group, # was *P* < 0.05, and ns was *P* > 0.05 vs. MI group. And *P* value was calculated by the long-rank test in (a), Student's *t*-test in (b), and post hoc comparisons in (c–f).

We first evaluated the effect of curcumin on the survival of MI mice. We found that 3 months after MI, the survival rate of MI mice in the untreated group was 46.67% (7/15), while that of MI mice treated with curcumin was 60.00% (9/15), but there was no significant difference in the 3-month overall survival between the two groups (*P* > 0.05, Figure 1). Next, we also examined the functional significance of curcumin on the histopathology and ventricular remodeling in MI mice at 3 months after MI. And there was no significant difference in the infarct size and lateral cardiomyocyte width (Figure 1) of MI mice, while the posterior and septal cardiomyocyte width in MI mice without any treatment was significantly higher than that of MI mice treated with curcumin (Figure 1). At the same time, the lateral and septal myocardial fibrosis in the border area of MI mice without any treatment was significantly higher than that of MI mice treated with curcumin (Figure 1), but there was no significant difference in lateral, posterior, and septal myocardial fibrosis in the remote area between the MI group and MI+Cur group (Figure 1). And qPCR analysis also showed that the expression levels of atrial natriuretic peptide (ANP), brain natriuretic peptide (BNP), and COL1A1 mRNA in the myocardium of MI group mice at 3 months post-MI were all significantly higher than those of mice in the MI+Cur group (Figure 1). Furthermore, data from echocardiography showed better LV function in MI mice treated with curcumin as compared with that in MI mice without any treatment; the LVDd and LVDs of mice in the MI group were significantly higher than those of mice in the MI+Cur group, whereas the EF and LVEF of mice in the MI group were significantly lower than those of mice in the MI+Cur group (Table 2).

### 3.2. Curcumin Inhibits Inflammation within One Week after MI

**Figure 2:**
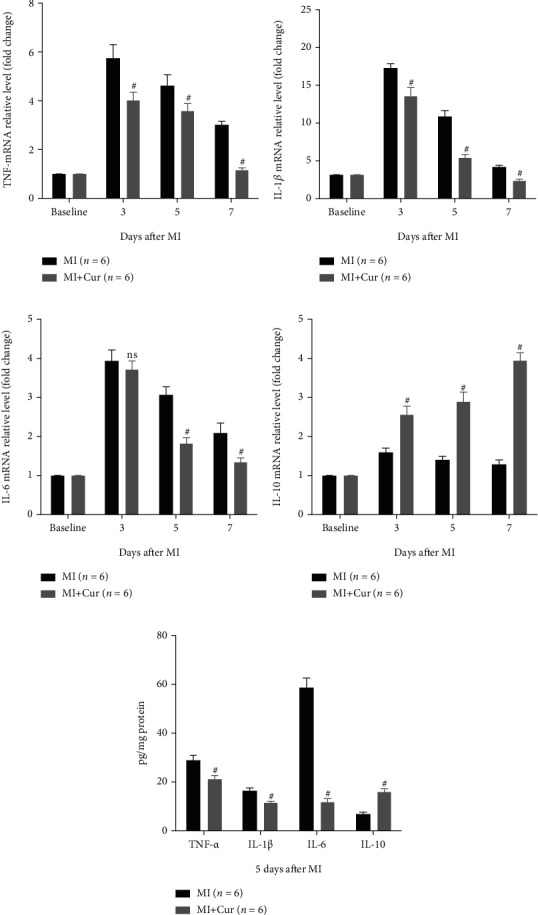
Analysis of inflammation-related cytokine mRNA and protein expression in homogenized hearts. Data were shown as the mean ± SEM. # was *P* < 0.05, and ns was *P* > 0.05 vs. the MI group. And *P* value was calculated by Student's *t*-test. Baseline was defined as the gene expression in a normal mouse myocardium.

Inflammation is one of the main causes of tissue injury and ventricular remodeling after MI [6, 21], and inhibiting early inflammation after MI will help tissue repair and reduce ventricular remodeling [22]. Here, we determined the expression of proinflammatory cytokines (TNF-*α*, IL-1*β*, and IL-6) and anti-inflammatory cytokines (IL-10) in the myocardium of MI mice at the first week post-MI. As shown in Figure 2, compared with baseline, proinflammatory cytokines (TNF-*α*, IL-1*β*, and IL-6) and anti-inflammatory cytokines (IL-10) are highly expressed in the first week after MI, but their dynamic changes are different. The expression of proinflammatory cytokines was the highest on the 3^rd^ day after MI and gradually decreased on the 5^th^ and 7^th^ days after MI, while the expression of inflammatory cytokines was the lowest on the 3^rd^ day after MI and gradually increased on the 5^th^ and 7^th^ days after MI. Importantly, compared with the MI group, the expression of proinflammatory cytokines was decreased, but the expression of anti-inflammatory cytokines was increased in the myocardium of MI mice in the MI+Cur group at the 3^rd^, 5^th^, and 7^th^ days post-MI.

### 3.3. Curcumin Suppresses M1 but Enhances M2 Macrophage Polarization In Vivo

**Figure 3:**
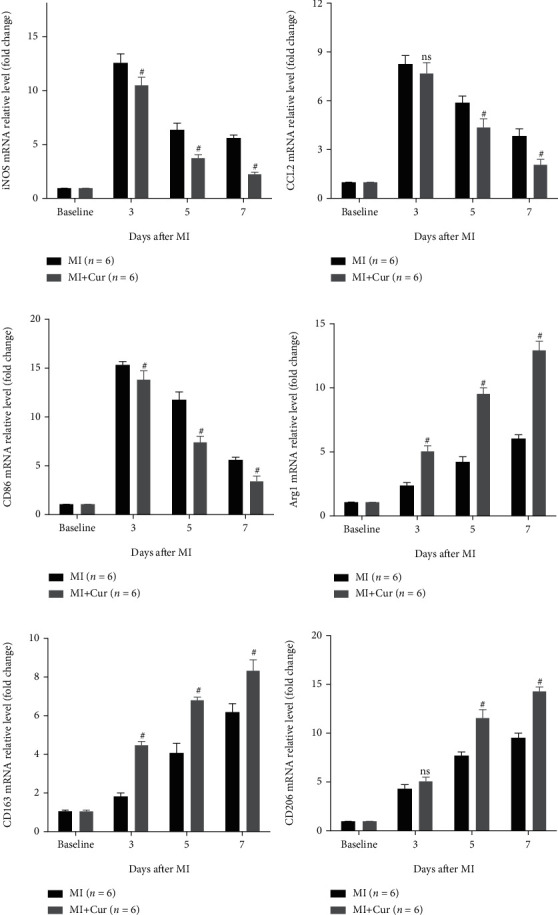
Curcumin decreases M1 macrophage marker expression (iNOS, CCL2, and CD86) and increases M2 marker mRNA expression (Arg1, CD163, and CD206) in homogenized hearts. Data were shown as the mean ± SEM. # was *P* < 0.05, and ns was *P* > 0.05 vs. the MI group. And *P* value was calculated by Student's *t*-test. Baseline was defined as the gene expression in a normal mouse myocardium.

**Figure 4:**
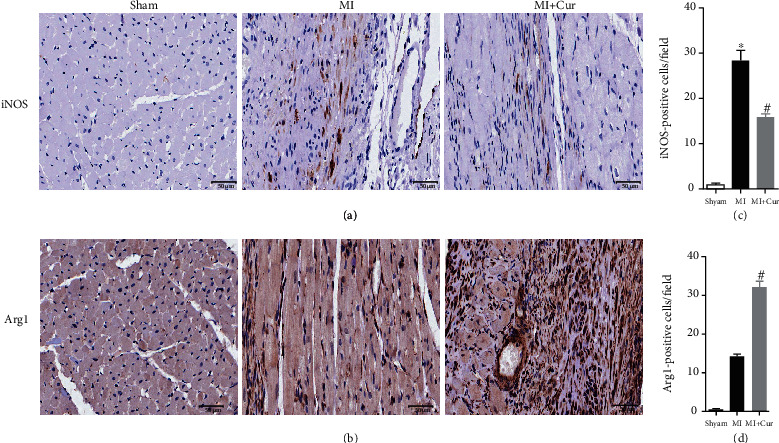
Effect of curcumin on M1 and M2 macrophage marker protein expression in the myocardium at 7 days post-MI. (a) Representative immunohistochemistry detection of M1 macrophage marker (iNOS) expression; (b) representative immunohistochemistry detection of M2 macrophage marker (Arg1) expression; (c) quantification of iNOS-positive cells; (d) quantification of Arg1-positive cells. Seven mice in each group. Data were shown as the mean ± SEM; ∗ was *P* < 0.05 vs. the sham group, and # was *P* < 0.05 vs. the MI group. And *P* value was calculated by Student's *t*-test.

**Figure 5:**
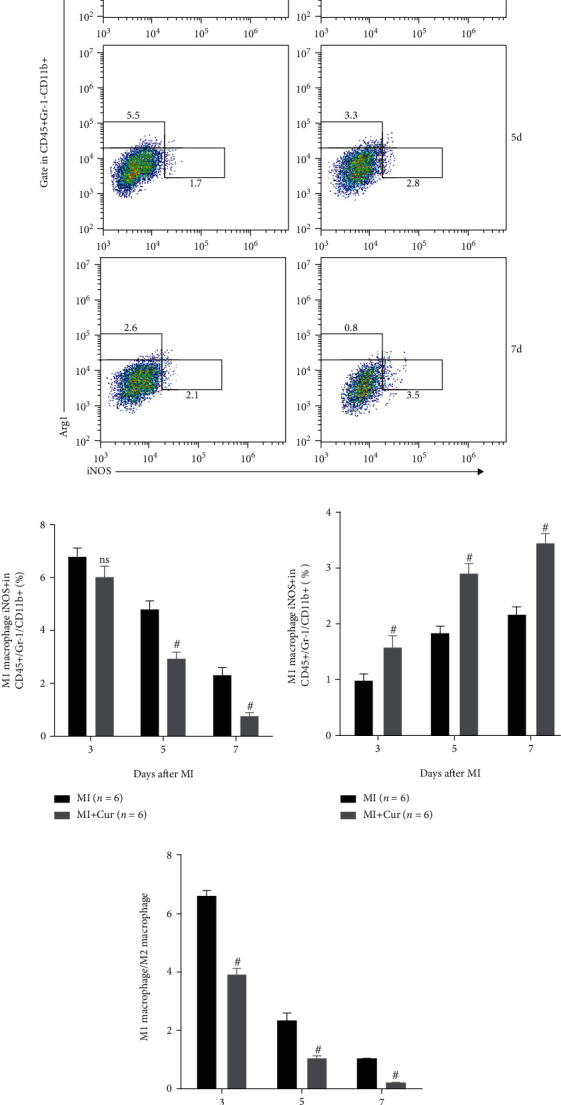
Curcumin decreases M1 macrophage and increases M2 macrophage in homogenized hearts. (a–d) Representative flow cytometry data of activated macrophages in the heart tissues of different groups (a), quantification of M1 (b) and M2 (c) macrophages, and calculation of the ratio of M1 and M2 macrophages (d). Data were shown as the mean ± SEM. # was *P* < 0.05 vs. the MI group. *P* value was calculated by Student's *t*-test.

Macrophage infiltration is the main cause and landmark pathological change of myocardial injury after MI, and it is polarized to M1 macrophages causing inflammation, while it is polarized to M2 macrophages inhibiting inflammation [23, 24]. Therefore, we studied the types of infiltrated macrophages in heart tissues of MI mice. Firstly, qPCR analysis indicated that (Figure 3) compared with baseline, M1 macrophage markers (iNOS, CCL2, and CD86) and M2 macrophage markers (Arg1, CD163, and CD206) are highly expressed in the first week after MI, but their dynamic changes are different. The expression of M1 macrophage markers was the highest on the 3^rd^ day after MI and gradually decreased on the 5^th^ and 7^th^ days after MI, while the expression of M2 macrophage markers was the lowest on the 3^rd^ day after MI and gradually increased on the 5^th^ and 7^th^ days after MI. Importantly, compared with the MI group, the expression of M1 macrophage markers was decreased, but the expression of M2 macrophage markers was increased in the myocardium of MI mice in the MI+Cur group at the 3^rd^, 5^th^, and 7^th^ days post-MI. Additionally, we also measured the expression of iNOS and Arg1 protein in the myocardium at 7 days post-MI, and we found that (Figure 4) the number of M1 macrophages (iNOS+F4/80+ cells) in the myocardium of MI mice without any treatment at 7 days post-MI was significantly higher than that in the myocardium of MI mice treated with curcumin at 7 days post-MI, while the number of M2 macrophages (Arg+F4/80+ cells) was significantly lower. In addition, we also prepared a single-cell suspension of heart tissue and then analyzed the proportion of different types of macrophages by flow cytometry; the data showed that (Figure 5) the proportion of M1 macrophages (CD45+Gr-1-CD11b+iNOS+ cells) was the highest on the 3^rd^ day after MI and gradually decreased on the 5^th^ and 7^th^ days after MI, while the proportion of M2 macrophages (CD45+Gr-1-CD11b+Arg+ cells) was the lowest on the 3^rd^ day after MI and gradually increased on the 5^th^ and 7^th^ days after MI. Importantly, compared with the MI group, the proportion of M1 macrophages was decreased, but the proportion of M2 macrophages was increased in the myocardium of MI mice in the MI+Cur group at the 3^rd^, 5^th^, and 7^th^ days post-MI.

### 3.4. Curcumin Regulates Bone Marrow Macrophage Polarization In Vitro

**Figure 6:**
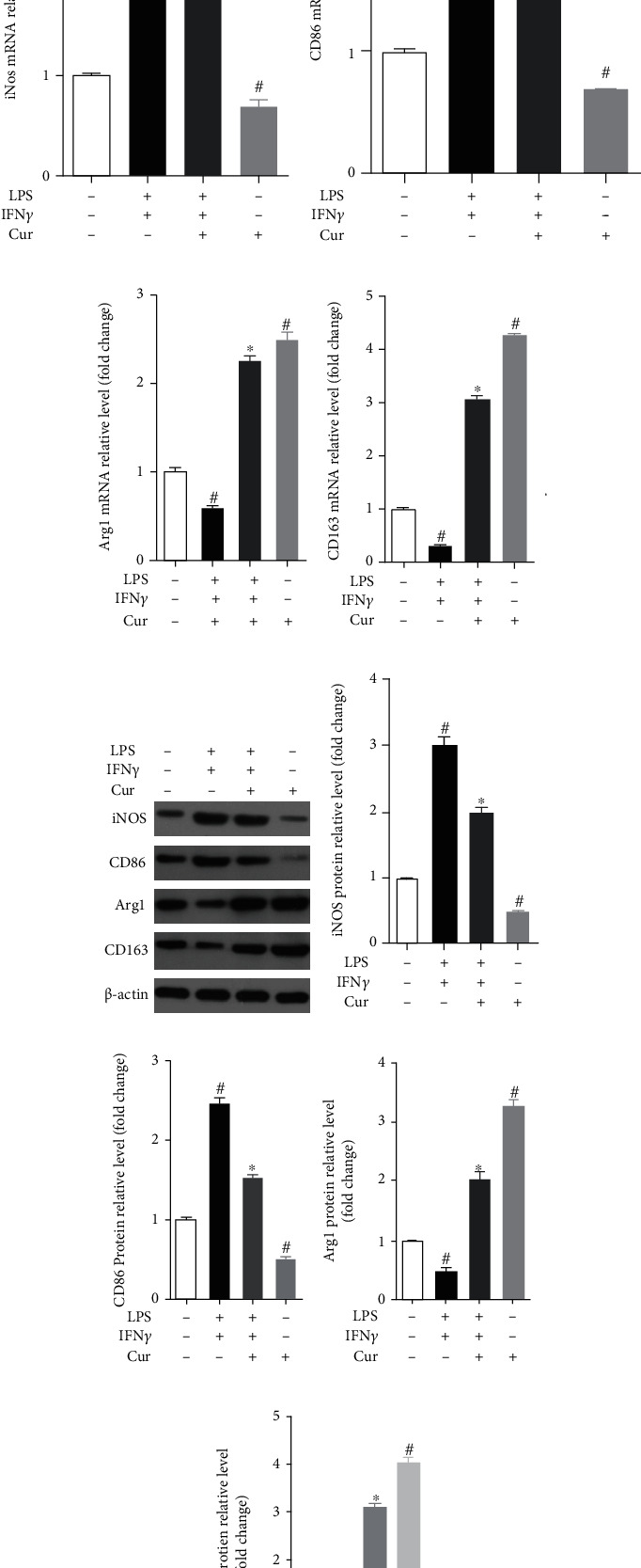
Effect of curcumin on LPS/IFN*γ*-induced M1 and M2 macrophage marker expression in BMMs. mRNA expression of iNOS (a), CD86 (b), Arg1 (c), and CD163 (d) in BMMs using qPCR after being stimulated with different stimuli. Representative protein bands (e) and quantification of the grayscale value of iNOS (f), CD86 (g), Arg1 (h), and CD163 (i) protein. Each experiment was repeated 3 times independently. Data were shown as the mean ± SEM. ∗ was *P* < 0.05 vs. the LPS+IFN*γ* stimulation group, and # was *P* < 0.05 vs. the unstimulated group. *P* value was calculated by post hoc comparisons.

In vitro, we isolated mouse bone marrow cells and then differentiated them into bone marrow macrophages (BMMs) and used LPS+INF*γ* to polarize BMMs to study the effect of curcumin on macrophage polarization. Firstly, the results of qPCR and immunoblotting analysis showed that (Figure 6) the expression of M1 macrophage markers (iNOS and CD86) increased and the expression of M2 macrophage markers (Arg1 and CD163) decreased in BMMs after being stimulated with LPS+IFN*γ*, but curcumin could reverse these changes. Interestingly, the expression of M1 macrophage markers (iNOS and CD86) decreased, and the expression of M2 macrophage markers (Arg1 and CD163) increased in BMMs after being stimulated with curcumin. Moreover, we also measured the concentration of some cytokines in the culture medium of BMMs after being stimulated with different stimuli and found that the concentration of proinflammatory cytokines (TNF-*α*, IL-1*β*, and IL-6) (Figures 6(a)–6(c)) increased after being stimulated with LPS+IFN*γ*, and curcumin decreased them. Oppositely, the concentration of anti-inflammatory cytokines (IL-10) (Figure 6(d)) was decreased after being stimulated with LPS+IFN*γ*, and curcumin could increase them.

### 3.5. Curcumin Modulates M1/M2 Macrophage Polarization Partly via AMPK

**Figure 7:**
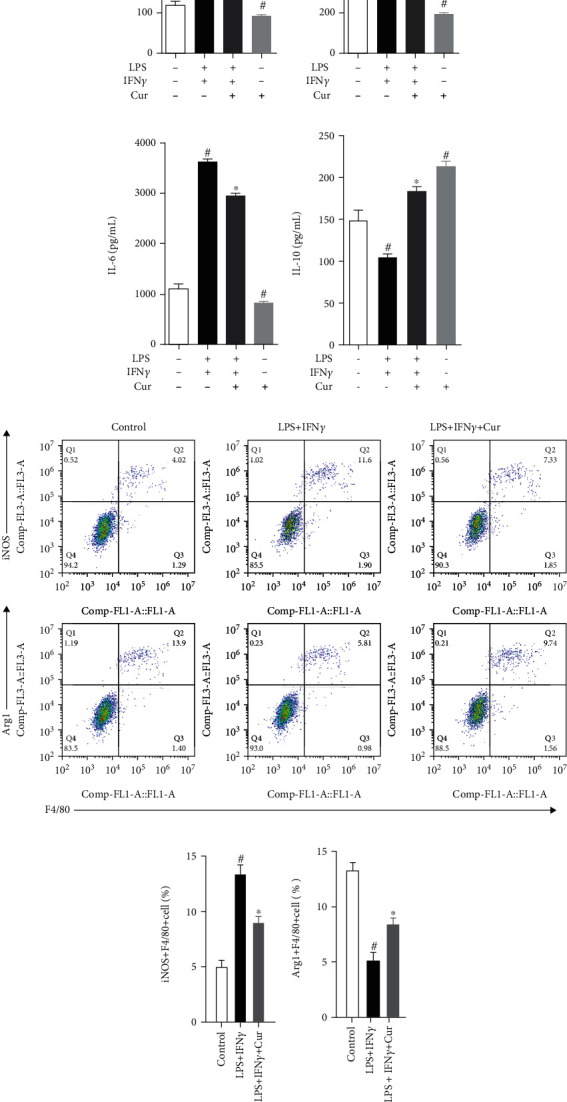
Effect of curcumin on LPS/IFN*γ*-induced macrophage polarization in BMMs. Concentration of TNF-*α* (a), IL-1*β* (b), IL-6 (c), and IL-10 (d) in cell culture medium of BMMs after being stimulated with different stimuli. Representative flow cytometry data of activated macrophages after being stimulated with different stimuli (e) and quantification of M1 (iNOS+F4/80+) (f) and M2 (Arg+F4/80+) (g) macrophages. Each experiment was repeated 3 times independently. Data were shown as the mean ± SEM. ∗ was *P* < 0.05 vs. the LPS+IFN*γ* stimulation group, and # was *P* < 0.05 vs. the unstimulated group. *P* value was calculated by post hoc comparisons.

**Figure 8:**
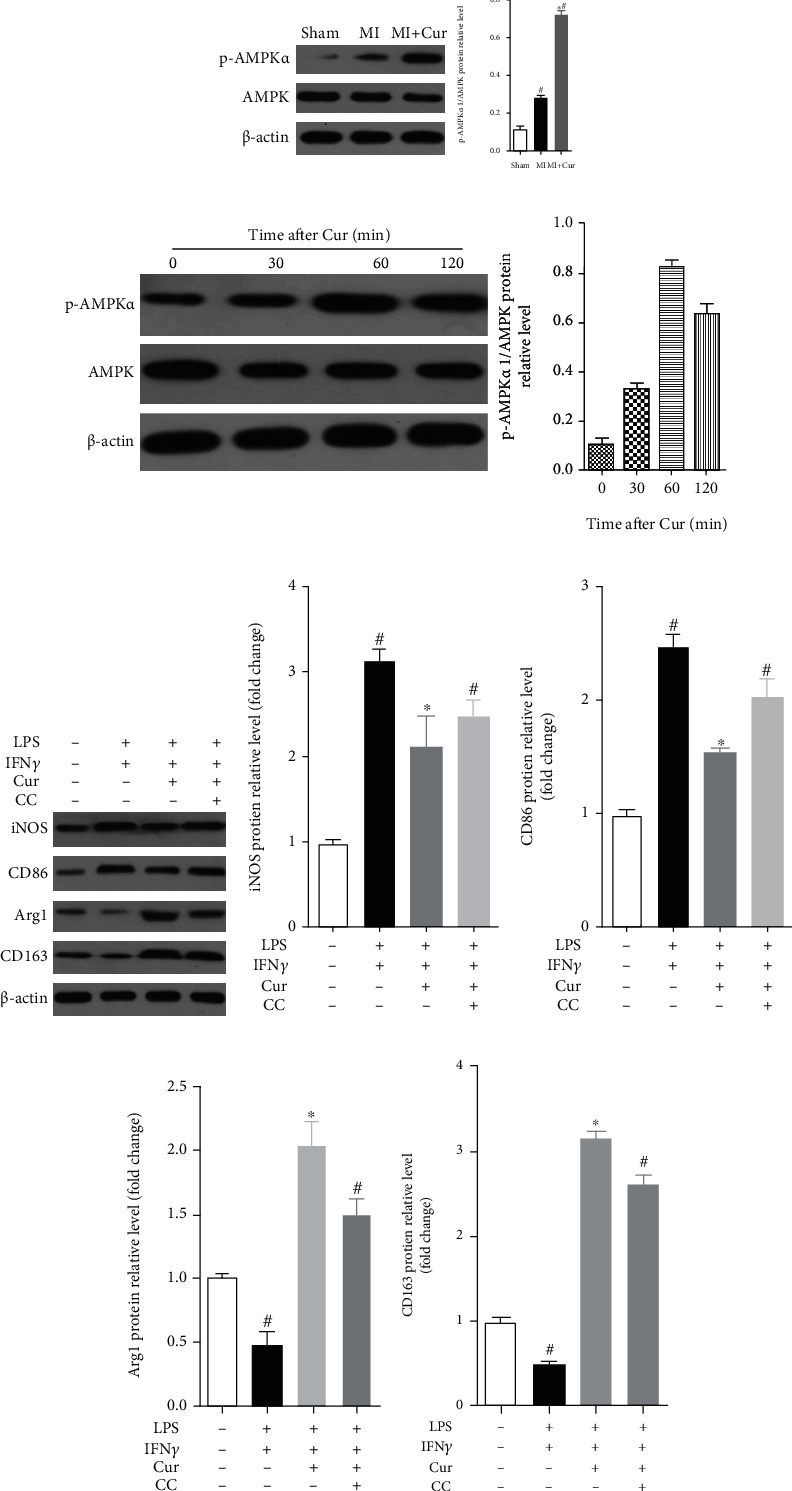
Curcumin modulates M1/M2 macrophage polarization partly via AMPK. (a) The expression of p-AMPK and AMPK protein in homogenized hearts at 7 days after MI: representative protein bands (left) and quantification of protein gray values (right), 7 mice in each group. Data were shown as the mean ± SEM. ∗ was *P* < 0.05 vs. the sham group, and # was *P* < 0.05 vs. the MI group. And *P* value was calculated by Student's *t*-test. (b) After being stimulated with curcumin for different times, p-AMPK and AMPK proteins in BMMs were expressed: representative protein bands (left) and quantification of protein gray values (right). Representative protein bands (c) and quantification of the grayscale value of iNOS (d), CD86 (e), Arg1 (f), and CD163 (g) protein. Each experiment was repeated 3 times independently. Data were shown as the mean ± SEM. ∗ was *P* < 0.05 vs. the LPS+IFN*γ* stimulation group, and # was *P* < 0.05 vs. the LPS+IFN*γ*+Cur stimulation group. *P* value was calculated by post hoc comparisons.

**Figure 9:**
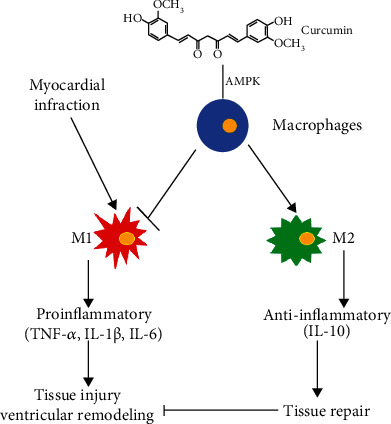
Schematic diagram of curcumin regulating macrophage polarization to inhibit MI-induced inflammation.

AMPK has been found to inhibit inflammation by mediating the polarization of macrophages [25, 26] and is the target of curcumin [27, 28]. Therefore, we hypothesized that AMPK also mediates curcumin-regulated macrophage polarization in the myocardium in MI mice. To test this hypothesis, we measured the expression of p-AMPK and AMPK protein in homogenized hearts at 7 days after MI and found that (Figure 7(a)) the expression of p-AMPK/AMPK protein increased in homogenized hearts of MI mice at 7 days after MI as compared with that of sham mice, and curcumin could decrease the elevated p-AMPK/AMPK protein expression in homogenized hearts of MI mice at 7 days after MI. In vitro, we first evaluated the expression of p-AMPK and AMPK protein in BMMs by curcumin and found that (Figure 6(b)) the phosphorylation of AMPK underwent an early upregulation and was characterized by the peaking stage reached at the first 60 minutes postcurcumin stimulation, and then, the production of p-AMPK in BMMs exhibited a decline. Therefore, we set the curcumin stimulation time at 60 minutes in the following experiment. As shown in Figure 7(c), the inhibitor of AMPK, Compound C (CC), reversed the inhibition of curcumin on iNOS (Figure 7(d)) and CD86 (Figure 7(e)) protein expression and reversed the elevation of curcumin on Arg1 (Figure 7(f)) and CD163 (Figure 7(g)) protein expression in BMMs after costimulating with LPS/IFN*γ*. Therefore, curcumin suppressed the MI-induced inflammation by modulating macrophage polarization partly via the AMPK pathway (Figures 8 and 9).

## 4. Discussion

In this study, we first found that curcumin attenuated myocardial remodeling improving cardiac function at 3 months after myocardial infarction, which was consistent with the previous study [13]. Importantly, we also found that curcumin not only suppressed myocardial inflammation but also decreased M1 but enhanced M2 macrophage polarization in the myocardium during the first 7 days post-MI. In vitro, curcumin also inhibited LPS/IFN*γ*-induced M1 macrophage polarization and promoted M2 macrophage polarization.

A series of physiological activities induced by sterile inflammation are essential for tissue repair after myocardial infarction. In the early stage of myocardial infarction, immune cells, including macrophages, are recruited to the infarct area and border area to eliminate dead cells, cell debris, and cytoplasmic matrix debris by releasing cytokines. Subsequently, by entering into the tissue repair phase, inflammation gradually resolved and scar formation occurred. Whether in the period of inflammation or in the period of inflammation remission, macrophages play an important role. Macrophages are the inflammatory cells with the longest residence time in the repair of myocardial tissue damage following MI injury [29, 30]. In the early stage of inflammation, it mainly plays the role of proinflammatory response and swallowing and promotes the regeneration and remodeling of granulation tissue in the middle and late stages of inflammation [31, 32].

It has been well known that macrophages have two phenotypes, namely, M1 macrophages and M2 macrophages. M1 macrophages can promote inflammation by secreting proinflammatory factors (IL-1*β*, IL-6, IL-12, and TNF-*α*), chemokines (MCP-1), and iNOS, and its specific marker is iNOS, while M2 macrophages exert anti-inflammatory effects by producing large amounts of anti-inflammatory factors (IL-10) and inhibiting the secretion of proinflammatory factors IL-12, IL-1, and TNF-*α*, and its specific marker is arginase 1 [23, 24]. The differentiation and activation of macrophages depend on specific growth and differentiation factors, receptors, signaling pathways, and transcription factors [33, 34]. Among them, cytokines secreted by T helper cells (Th) focus on the polarization of macrophages. According to the difference of secreted cytokines, Th cells are divided into two types, Th1 and Th2. Th1 cells are characterized by secreting cytokines such as IFN*γ*, IL-1, IL-2, and TNF-*α*, which polarize macrophages into M1 macrophages. Th2 cells secrete IL-4, IL-5, IL-6, IL-10, IL-13, and so on, which polarize macrophages into M2 macrophages [33, 34]. Therefore, in the present study, we evaluated the effect of curcumin on the expression of myocardial cytokines and macrophage phenotype marker in MI mice, and these results, as well as subsequent flow cytometry analysis data, showed that curcumin inhibited early myocardial inflammation and regulated macrophage polarization after MI.

To further confirm that curcumin inhibits inflammation and regulates the polarization of macrophages, we prepared mouse bone marrow macrophages and found that curcumin not only inhibited LPS/IFN*γ*-induced inflammation but also inhibited the polarization of BMMs to M1 macrophages but promotes the polarization of BMMs to M2 macrophages. Similarly, previous studies have shown that curcumin can promote the polarization of M2 macrophages in autoimmune myocarditis [35] and daunorubicin induced nephrotoxicity [36] in rats and macrophage stable cell lines [35]. Some data reveals some of the molecular mechanisms of curcumin regulating macrophage polarization, including promoting IL-4 and IL-13 (M2 macrophage activator) secretion [17]and inhibiting IL-12 and TNF production (M2 macrophage activator) [37]. Here, we focused on the AMPK signaling pathway, and we found that curcumin inhibits the activation of the AMPK signaling pathway in the MI mouse myocardium, and the AMPK inhibitor attenuates curcumin's promotion of M2 macrophage polarization in vitro.

AMPK not only is a cell stress sensor but also plays an important role in inhibiting inflammatory response, and the phosphorylation of AMPK protein is a sign of activation of the AMPK signaling pathway [38, 39]. At the same time, previous studies have shown that AMPK has been found to inhibit inflammation by mediating the polarization of macrophages [25, 26] and is the target of curcumin [27, 28]. However, our data just showed that the AMPK inhibitor partly attenuated curcumin's promotion of M2 macrophage polarization in vitro, indicating that AMPK is not the only target for curcumin to regulate macrophage polarization. In conclusion, curcumin suppressed the MI-induced inflammation by modulating macrophage polarization partly via the AMPK pathway.

## Figures and Tables

**Table 1:** Sequence of qPCR primers.

Gene	Sequence (5′-3′)
ANP	Forward: GTGCGGTGTCCAACACAGAT
	Reverse: TCCAATCCTGTCAATCCTACCC
BNP	Forward: GAGGTCACTCCTATCCTCTGG
	Reverse: GCCATTTCCTCCGACTTTTCTC
COL1A1	Forward: GCTCCTCTTAGGGGCCACT
	Reverse: ATTGGGGACCCTTAGGCCAT
TNF-*α*	Forward: CCTGTAGCCCACGTCGTAG
	Reverse: GGGAGTAGACAAGGTACAACCC
IL-1*β*	Forward: ACAGCAAAAGTTACGGTAGCAG
	Reverse: ATGGGTTCCCCAATGACTTCA
IL-6	Forward: CCTGAACCTTCCAAAGATGGC
	Reverse: TTCACCAGGCAAGTCTCCTCA
IL-10	Forward: CTTACTGACTGGCATGAGGATCA
	Reverse: GCAGCTCTAGGAGCATGTGG
iNOS	Forward: GTTCTCAGCCCAACAATACAAGA
	Reverse: GTGGACGGGTCGATGTCAC
CD86	Forward: TCAATGGGACTGCATATCTGCC
	Reverse: GCCAAAATACTACCAGCTCACT
Arg1	Forward: CTCCAAGCCAAAGTCCTTAGAG
	Reverse: GGAGCTGTCATTAGGGACATCA
CD163	Forward: GGTGGACACAGAATGGTTCTTC
	Reverse: CCAGGAGCGTTAGTGACAGC
*β*-Actin	Forward: AGCCCATCCTTCGAGTACAAA
	Reverse: TCTTGGTGCGATAACTGGTGG

**Table 2:** Effect of curcumin on echocardiographic parameters of MI mice at 3 months after myocardial infarction.

		Sham (*n* = 6)	MI (*n* = 7)	MI+Cur (*n* = 9)
Heart rate (bmp)		446 ± 18	468 ± 12	472 ± 15
LVDd (mm)		4.08 ± 0.05	5.62 ± 0.13^∗^	5.36 ± 0.11^∗^^#^
LVDs (mm)		3.12 ± 0.03	5.62 ± 0.15^∗^	4.93 ± 0.18^∗^^#^
FS (%)		26.0 ± 1.5	6.9 ± 1.0^∗^	11.2 ± 1.8^∗^^#^
LVEF (%)		52.3 ± 0.5	13.8 ± 1.5^∗^	21.23 ± 2.3^∗^^#^
WThd, septum (mm)	Remote	0.98 ± 0.08	0.85 ± 0.15^∗^	0.90 ± 0.12^∗^
	Border	0.92 ± 0.05	0.43 ± 0.09^∗^	0.44 ± 0.12
WThd, free wall (mm)	Remote	0.91 ± 0.03	1.02 ± 0.08^∗^	1.01 ± 0.07
	Border	1.01 ± 0.06	0.48 ± 0.11^∗^	0.51 ± 0.09
WThs, septum (mm)	Remote	1.12 ± 0.15	0.85 ± 0.08^∗^	1.05 ± 0.08^∗^^#^
	Border	1.11 ± 0.08	0.49 ± 0.06^∗^	0.52 ± 0.11
WThs, free wall (mm)	Remote	1.15 ± 0.07	1.08 ± 0.05	1.14 ± 0.07
	Border	1.18 ± 0.09	0.48 ± 0.02^∗^	0.57 ± 0.10^∗^^#^

Data were shown as the mean ± SEM. ∗ was *P* < 0.05 vs. the sham group, and # was *P* < 0.05 vs. the MI group. And *P* value was calculated by post hoc comparisons. LVDd: left ventricular (LV) dimension at end diastole; LVDs: left ventricular (LV) dimension at end systole; FS: fractional shortening; LVEF: left ventricular (LV) ejection fraction; WThd: wall thickness at end diastole; WThs: wall thickness at end systole.

## Data Availability

The datasets used and/or analyzed during the present study are available from the corresponding author on reasonable request.
